# Hospital-Based Investigation of Acute Respiratory Infections in Children Under Five: Epidemiology, Seasonality, and Co-infections

**DOI:** 10.7759/cureus.96006

**Published:** 2025-11-03

**Authors:** Baijayantimala Mishra, Diksha Mohapatra, Sailendra Panda, Debashis Santra, Madhab Charan Mandal, Prabhudutta Mamidi, Bhagirathi Dwibedi, Rashmi Ranjan Das, Sunita Das, Prasanta R Mohapatra, Bijayini Behera, Krishna M Gulla, Tapas Kumar Som

**Affiliations:** 1 Department of Microbiology, All India Institute of Medical Sciences, Bhubaneswar, IND; 2 State-Level Viral Research and Diagnostic Laboratory (VRDL), Department of Microbiology, All India Institute of Medical Sciences, Bhubaneswar, IND; 3 Department of Pediatrics, All India Institute of Medical Sciences, Bhubaneswar, IND; 4 Department of Pulmonary Medicine and Critical Care, All India Institute of Medical Sciences, Bhubaneswar, IND; 5 Department of Neonatology, All India Institute of Medical Sciences, Bhubaneswar, IND

**Keywords:** co-infection, multiplex pcr, respiratory infection, respiratory pathogen, under five children

## Abstract

Background: Acute respiratory infections (ARIs) remain a leading cause of childhood morbidity and mortality in low-resource settings. This hospital-based observational study investigated the etiology, seasonal trends, and co-infections of respiratory pathogens using the multiplex polymerase chain reaction (PCR) panel among children under five from Eastern India.

Study design: From January to December 2024, 209 samples (nasopharyngeal swabs/throat swabs or bronchoalveolar lavage) were collected from children under five who visited a tertiary healthcare center at Bhubaneswar, Odisha, India, with ARI/severe ARI (SARI). Multiplex PCR was used to identify the pathogens. All the data were recorded in a pre-designed case record form and analyzed using appropriate statistical tests.

Result: Overall, 59.8% (125/209) tested positive for viral pathogens, predominantly human rhinovirus (HRV, 21.05%) and respiratory syncytial virus (RSV, 16.26%). Bacterial pathogens were detected in 45.45% (95/209), primarily *Haemophilus influenzae* (11%) and *Klebsiella pneumoniae* (7.65%). Viral co-infections occurred in 6.69% (14/209), with HRV-RSV being the most common, while bacterial co-infections (8.61%) frequently involved *Streptococcus pneumoniae* and *H. influenzae*. Seasonal peaks for viral infections occurred post-monsoon (October), whereas bacterial infections peaked during monsoon (September). ARI cases showed higher odds of bacterial etiology (OR 1.04, P = 0.87), whereas SARI cases showed higher odds of viral etiology (OR 0.65, P = 0.04).

Conclusion: The findings underscore HRV and *H. influenzae* as dominant agents, highlighting the need for region-specific surveillance, improved diagnostics, and targeted vaccination strategies to mitigate ARI burden in children under five.

## Introduction

Acute respiratory infections (ARIs) are classified into upper and lower respiratory tract infections. It is a significant public health concern that affects individuals of all ages, particularly younger children [1]. Pediatric ARIs are more likely to be caused by viruses than bacteria; however, identifying a particular respiratory virus by symptomatology is challenging, as respiratory viral infections often have overlapping symptoms [2]. As per the World Health Organization (WHO), every year, around 3-5 million cases of ARIs are reported globally, with ARI-related deaths ranging from 290,000 to 650,000, and 99% of the cases are reported in under-five children in developing countries [3]. According to the National Family Health Survey-5 (NFHS-5) conducted between 2019 and 2021, 68.5% of children under five years of age in India reportedly experience symptoms of ARI, while in Eastern India, the prevalence is 66.8% among urban children and 62.23% among rural children [4,5].

Seasonality, geography, and occupation considerably impact the prevalence and circulation of respiratory pathogens [6]. Eastern India shares its international borders with Nepal, Bhutan, and Bangladesh, which heightens the risk of cross-border transmission [7-9]. The warm and humid climate of the region and frequent flooding from rivers draining into the Bay of Bengal cause waterlogging and aid aerosol transmission, favoring the survival of viruses like influenza virus (IFV) and respiratory syncytial virus (RSV) [10,11]. In developing countries, a child typically experiences 6-8 episodes of ARIs annually. This accounts for a significant portion of the pediatric population, making up 30%-50% of outpatient visits and 20%-30% of admissions [12,13]. Globally, respiratory infections, mainly pneumonia, cause 20% of deaths in under-five children, rising to 35%-40% when neonatal pneumonia is included. As per the Indian mortality statistics, 140,600 children aged 1-59 months died due to pneumonia, corresponding to a mortality rate of approximately 4.2 per 1000 live births [14].

Along with seasonal patterns and pathogen transmission, low socioeconomic status, low literacy rate, suboptimal breastfeeding, malnutrition, inadequate immunization coverage, and use of cooking fuel instead of liquified petroleum gas have been attributed as some of the risk factors contributing to the increased burden of ARIs among children [15,16]. Recent advances in diagnostic techniques, especially multiplex polymerase chain reaction (PCR) assays, have improved pathogen detection accuracy and identification of co-infections. However, comprehensive data on the distribution, seasonality, and co-infection patterns of respiratory pathogens among under-five children in Eastern India remain scarce. Therefore, the present hospital-based observational study was conducted to investigate the etiology, seasonal trends, and frequency of mono-infection and co-infection of respiratory pathogens in under-five children in Eastern India using a multiplex PCR panel.

## Materials and methods

Study design

A retrospective hospital-based observational study was conducted at a tertiary healthcare center in Bhubaneswar, Odisha, India, in children under five who presented with symptoms of ARI and severe acute respiratory infections (SARI) to the pediatrics outpatient department (OPD) or were admitted to the inpatient department (IPD) from January to December 2024.

A standardized case report form (CRF) was used to collect each patient’s personal, demographic, clinical, and epidemiological details. The study followed WHO guidelines for sample collection, transportation, and storage [17]. Samples (nasal swab/throat swab, nasopharyngeal or bronchoalveolar lavage) were collected by trained medical staff and transported to the State-Level Virus Research and Diagnostic Laboratory (VRDL), Department of Microbiology, All India Institute of Medical Sciences (AIIMS), Bhubaneswar, and maintained in a cold chain for investigation.

Ethics approval

The study received approval from the Institutional Ethics Committee of AIIMS Bhubaneswar (Ref. number: EMF 39/202, dated February 12, 2024).

Subject enrolment and case definition

All under-five children who presented with ARI/SARI were enrolled as per the WHO case definitions in the study, and samples were collected.

ARI was diagnosed in patients who presented to the OPD with an acute onset of any two of the symptoms, such as fever, cough, rhinorrhea, nasal congestion, or shortness of breath. To diagnose SARI, the WHO case definition was used, which encompasses cases presenting recent onset of fever (≥38°C) and cough starting within a week or diagnosis of acute lower respiratory infections (ALRIs), such as pneumonia, bronchitis, or bronchiolitis requiring hospitalization [18,19].

Inclusion and exclusion criteria

All children under five years of age presenting with symptoms suggestive of ARI or SARI, as per WHO case definitions, were included in the study. Children were enrolled prior to the initiation of antimicrobial therapy to ensure accurate pathogen detection. Duplicate or repeat samples from the same episode of illness and children who had received antibiotics for more than 48 hours before sample collection were excluded from the study.

Molecular detection

The extraction of viral ribonucleic acid (RNA) from suspected clinical samples was performed utilizing the QIAamp Viral RNA Mini Kit (Ref. 52904, Qiagen, Hilden, Germany), following the manufacturer's guidelines. Deoxyribonucleic acid (DNA) extraction was performed using the QIAamp DNA Blood mini kit (Ref. 57104, Qiagen, Hilden, Germany), following the manufacturer's guidelines. The extracted viral RNA was subjected to identify a panel of respiratory viruses using the TRU PCR respiratory viral pathogen panel kit (Ref. 3B287-V, 3BBlackBio Biotech India Ltd., Bhopal, India) following the manufacturer’s guidelines on a real-time PCR platform, where the cutoff cycle threshold (Ct) value for PCR positivity was 33.

Similarly, the extracted DNA was used to identify different respiratory bacterial pathogens using TRU PCR respiratory bacterial pathogen panel kit (Ref. 3B287-B, 3BBlackBio Biotech India Ltd., Bhopal, India) following manufacturer’s guidelines on Bio-Rad CFX 96 (Bio-Rad Laboratories India Pvt. Ltd, India) and Quantstudio 5 Dx (Thermo Fisher Scientific, USA) real-time PCR platforms, where the cutoff Ct value for PCR positivity was 36.

Statistical analysis

Patient details were entered into the Department of Health Research (DHR)-Virus Research Diagnostic Laboratory (VRDL) network database portal as a part of the routine surveillance activity of the State-Level VRDL, AIIMS, Bhubaneswar. The data were later extracted using Microsoft Excel (Microsoft Corp., Redmond, WA, USA) and then loaded into IBM SPSS Statistics for Windows, Version 21.0 (Released 2012; IBM Corp., Armonk, NY, USA) for statistical analysis. The odds ratio (OR) for comparative analysis of quantitative variables was obtained using Jamovi 2.6.44 software.

## Results

Patient characteristics

**Figure 1:**
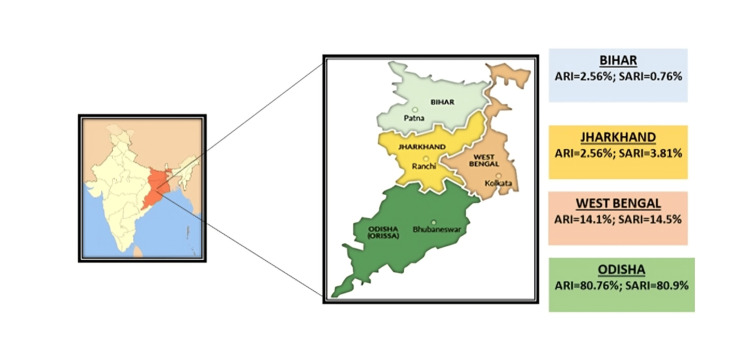
Distribution of ARI and SARI cases in different states of Eastern India. ARI: acute respiratory infections, SARI: severe acute respiratory infections.

A total of 209 respiratory samples from children under five diagnosed with ARI and SARI were received at the State-Level VRDL, Department of Microbiology, AIIMS, Bhubaneswar, during the study period. One hundred sixty-nine samples were from the state of Odisha, 30 were from West Bengal, 7 were from Jharkhand, and 3 were from Bihar. The majority (148, 70.81%) were male and 61 (29.18%) were female. Of the 209 patients, 78 (37.32%) had ARI, and 131 (62.67%) had SARI. The details of ARI/SARI cases included in the study are elaborated in Table 1, and Figure 1 illustrates the distribution of ARI and SARI cases in different states of Eastern India.

**Table 1:** Demographic and clinical details of the study population. NP: nasopharyngeal, BAL: bronchoalveolar lavage.

Features	ARI (n = 78) (%)	SARI (n = 131) (%)
Demographic details		
Odisha (n = 169)	63/78 (80.76)	106/131 (80.9)
West Bengal (n = 30)	11/78 (14.1)	19/131 (14.5)
Jharkhand (n = 7)	02/78 (2.56)	05/131 (3.81)
Bihar (n = 3)	02/78 (2.56)	01/131 (0.76)
Patient characteristics		
Mean age (in months)	17.28 ± 11.45	20.77 ± 12.46
Male	62/78 (79.48)	86/131 (65.64)
Female	16/78 (20.51)	45/131 (34.35)
Mean duration of illness (in days)	11.8 ± 10.09	13.44 ± 11.68
Mean duration of hospitalization (in days)	6.17 ± 15.23	9.78 ± 17.11
Age group		
0-2 months (n = 24)	08 (10.25)	16 (12.21)
2 months-1 year (n = 112)	47 (60.25)	65 (49.61)
1-3 years (n = 38)	14 (17.94)	24 (18.32)
3-5 years (n = 35)	09 (11.53)	26 (19.84)
Sample collected		
NP swab	69/78 (88.46)	113/131 (86.25)
Throat swab	02/78 (2.56)	02/131 (1.52)
BAL	07/78 (8.97)	16/131 (12.21)
Clinical features		
Fever	54/78 (69.23)	87/131 (66.4)
Chills	01/78 (1.28)	01/131 (0.76)
Rigor	02/78 (2.56)	01/131 (0.76)
Rhinorrhea	29/78 (37.17)	29/131 (22.13)
Sore throat	10/78 (12.82)	23/131 (17.55)
Cough	57/78 (73.07)	85/131 (64.88)
Breathlessness	32/78 (41.02)	69/131 (52.67)
Wheezing	01/78 (1.28)	98/131 (74.80)
Crepitation	0/78	42/131 (32.06)
Headache	10/78 (12.82)	0/131
Irritability	16/78 (20.51)	05/131 (3.81)
Altered sensorium	02/78 (2.56)	03/131 (2.29)
Seizure	03/78 (3.84)	05/131 (3.81)
Abdominal pain	01/78 (1.28)	02/131 (1.52)
Vomiting	05/78 (6.41)	08/131 (6.10)
Diarrhea	01/78 (1.28)	0/131
Malaise	03/78 (3.84)	01/131 (0.76)
Arthralgia	02/78 (2.56)	0/131
Myalgia	02/78 (2.56)	0/131
Maculopapular rash	04/78 (5.12)	0/131
Eschar	01/78 (1.28)	0/131
Jaundice	02/78 (2.56)	03/131 (2.29)
Mortality (n = 4)	2 (2.56)	2 (1.52)

Viral pathogens detected in ARI/SARI cases

Of the 209 samples, 125 (59.8%) were positive for viral pathogens, in which HRV (44, 21.05%) was most commonly detected, followed by RSV A and B (34, 16.26%). HRV was most common in the 1- 3 years age group (11, 28.94%), followed by 3-5 years (8, 22.85%) and 0-2 months (5, 20.83%), whereas RSV A and B was more prevalent in the 2 months-1 year age group (24, 21.42%).

In ARI cases, RSV A and B (19, 24.35%) were the most common, followed by HRV (11, 14.10%). Similarly, in SARI cases, HRV (33, 25.19%) was the most common, followed by RSV A and B (15, 11.45%) and human parainfluenza virus (HPIV) (10, 7.63%): HPIV-1 (2), HPIV-2 (4), and HPIV-3 (4) (Table 2).

**Table 2:** Viral pathogens detected in ARI/SARI patients. ARI: acute respiratory infections, SARI: severe acute respiratory infections, HRV: human rhinovirus, RSV: respiratory syncytial virus, HPIV: human parainfluenza virus, HMPV: human metapneumovirus, HAdV: human adenovirus, HBoV: human bocavirus, HCoV: human coronavirus.

Viral pathogens	Total (n= 209) (%)	ARI (n = 78) (%)	SARI (n = 131) (%)
HRV	44 (21.05)	11 (14.10)	33 (25.19)
RSV A & B	34 (16.26)	19 (24.35)	15 (11.45)
HPIV	15 (7.17)	5 (6.41)	10 (7.63)
Influenza A	13 (6.22)	8 (10.25)	5 (3.81)
HMPV	9 (4.3)	1 (1.28)	8 (6.10)
Influenza B	3 (1.43)	0	3 (2.29)
HAdV	3 (1.43)	1 (1.28)	2 (1.52)
HBoV	2 (0.95)	0	2 (1.52)
HCoV	1 (0.47)	1 (1.28)	0
Enterovirus	1 (0.47)	0	1 (0.76)
Total	125 (59.8)	47 (60.25)	81 (61.83)

Co-infection of viral pathogens detected in ARI/SARI cases

**Figure 2:**
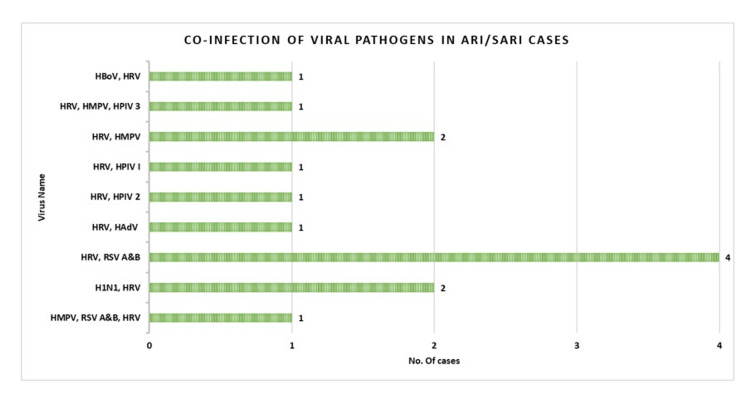
Co-infection of viral pathogens detected in ARI/SARI cases. ARI: acute respiratory infections, SARI: severe acute respiratory infections, HRV: human rhinovirus, RSV: respiratory syncytial virus, HPIV: human parainfluenza virus, HMPV: human metapneumovirus, HAdV: human adenovirus, HBoV: human bocavirus, HCoV: human coronavirus.

A total of 14 (6.69%) children tested positive for more than one viral pathogen and in all the co-infections, in which HRV was the most commonly detected pathogen. The highest co-infection of four (1.91%) cases was recorded with HRV and RSV A and B, followed by two (0.95%) cases of HRV and human metapneumovirus (HMPV) and two (0.95%) cases of HRV and H1N1. Other notable co-infections were with human bocavirus (HBoV), HMPV, HPIV-1, HPIV-2, HPIV-3, human adenovirus (HAdV), RSV A and B, and H1N1 (Figure 2).

Bacterial pathogens detected in ARI/SARI cases

Of 209 samples, 95 (45.45%) tested positive for bacterial pathogens, in which *H. influenzae *(23, 11%) was the most commonly detected, followed by *Klebsiella pneumoniae* (16, 7.65%) and *Streptococcus pneumoniae* (16, 7.65%). *H. influenzae* (1, 4.16%), *K. pneumoniae *(1, 4.16%), *Pseudomonas aeruginosa* (1, 4.16%), and *Staphylococcus aureus* (1, 4.16%) were detected in the 0-2 months age group. *H. influenzae* was the most prevalent in the 2 months-1 year age group (13, 11.6%) and 1-3 years (6, 15.78%), while *Mycoplasma pneumoniae* (6, 17.14%) was more prevalent in the 3-5 years age group.

In ARI cases, *H. influenzae* (9, 11.53%) was the most common, followed by* K. pneumoniae* (6, 7.69%), *P. aeruginosa* (5, 6.41%), and *Acinetobacter baumannii* (5, 6.41%). Similarly, in SARI cases, *H. influenzae *(14, 10.68%) was the most common, followed by *S. pneumoniae* (12, 9.16%) (Table 3).

**Table 3:** Bacterial pathogens detected in ARI/SARI patients. ARI: acute respiratory infections, SARI: severe acute respiratory infections.

Bacterial pathogens	Total (n = 209) (%)	ARI (n = 78) (%)	SARI (n = 131) (%)
Haemophilus influenzae	23 (11)	9 (11.53)	14 (10.68)
Klebsiella pneumoniae	16 (7.65)	6 (7.69)	10 (7.63)
Streptococcus pneumoniae	16 (7.65)	4 (5.12)	12 (9.16)
Mycoplasma pneumoniae	12 (5.74)	3 (3.84)	9 (6.87)
Pseudomonas aeruginosa	10 (4.78)	5 (6.41)	5 (3.81)
Staphylococcus aureus	7 (3.34)	3 (3.84)	4 (3.05)
Acinetobacter baumannii	6 (2.87)	5 (6.41)	1 (0.76)
Moraxella catarrhalis	5 (2.39)	0	5 (3.81)
Total	95 (45.45)	35 (44.87)	60 (45.80)

Co-infection of bacterial pathogens detected in ARI/SARI cases

**Figure 3:**
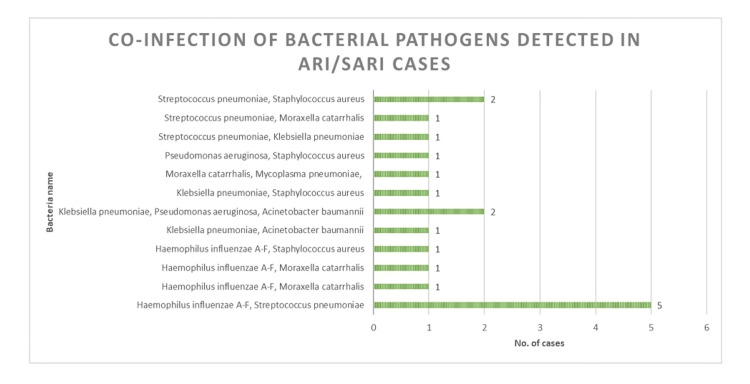
Co-infection of bacterial pathogens detected in ARI/SARI cases. ARI: acute respiratory infections, SARI: severe acute respiratory infections.

A total of 18 (8.61%) patients tested positive for more than one bacterial pathogen. The highest co-infection in five (2.39%) cases was detected with *H. influenzae* A-F and *S. pneumoniae*. In two (0.95%) cases, co-infection of three bacterial pathogens was detected, namely, *K. pneumoniae*, *P. aeruginosa*, and *A. baumanii*. *S. pneumoniae* (9, 4.31%) was the most commonly detected co-infection pathogen, followed by *H. influenzae* A-F (8, 3.82%), *S. aureus* (5, 2.39%), and *K. pneumoniae* (5, 2.39%) (Figure 3).

Comparative analysis of clinical features of ARI cases detected with viral pathogens, bacterial pathogens, and co-infections with viral and bacterial pathogens

The comparative analysis of clinical features of ARI cases detected with a viral pathogen and bacterial pathogen alone versus those with viral-bacterial co-infections is presented in Table 4. The OR on comparing clinical features of ARI cases detected positive for viral pathogen and viral-bacterial co-infection was 0.91 (95% CI 0.55-1.48, I^2^ = 0%, p = 0.69), while the OR on comparing clinical features of ARI cases detected positive for bacterial pathogens and viral-bacterial co-infection was 1.04 (95% CI 0.64-1.69, I^2^ = 0%, p = 0.87).

**Table 4:** Comparative analysis of clinical features of ARI cases detected with viral pathogens, bacterial pathogens, and viral-bacterial co-infection.

Clinical features	Viral (n = 29) (%)	Bacterial (n = 28) (%)	Viral-bacterial co-infection (n = 14) (%)	OR (95% CI), viral vs. co-infection	OR (95% CI), bacterial vs. co-infection
Fever	20 (68.96)	20 (71.17)	10 (71.42)	0.89 (0.22, 3.61)	1.00 (0.24, 4.14)
Chills	1 (3.44)	0	0	1.53 (0.06, 39.86)	-
Rigor	2 (6.89)	0	0	2.64 (0.12, 58.66)	-
Rhinorrhea	8 (27.58)	11 (39.28)	6 (42.85)	0.51 (0.13, 1.93)	0.86 (0.23, 3.17)
Sore throat	2 (6.89)	4 (14.02)	1 (7.14)	0.96 (0.08, 11.61)	2.17 (0.22, 21.46)
Cough	19 (65.51)	22 (78.57)	11 (78.57)	0.52 (0.12, 2.30)	1.00 (0.21, 4.78)
Breathlessness	15 (51.72)	10 (35.71)	7 (50)	1.07 (0.30, 3.84)	0.56 (0.06, 41.34)
Wheezing	1 (3.44)	2 (7.14)	0	1.53 (0.06, 39.86)	2.74 (0.12, 60.92)
Crepitation	0	1 (3.57)	0	-	1.58 (0.06, 41.34)
Headache	3 (10.34)	3 (10.71)	2 (14.28)	0.69 (0.10, 4.70)	0.72 (0.11, 4.90)
Irritability	7 (24.13)	2 (7.14)	2 (14.28)	1.91 (0.34, 10.68)	0.46 (0.06, 3.68)
Altered sensorium	0	0	0	-	-
Seizure	1 (3.44)	2 (7.14)	1 (7.14)	0.46 (0.03, 8.02)	1.00 (0.08, 12.07)
Abdominal pain	0	1 (3.57)	0	-	1.58 (0.06, 41.38)
Vomiting	0	4 (14.02)	1 (7.14)	0.15 (0.01, 3.99)	2.17 (0.22, 21.46)
Diarrhea	0	1 (3.57)	0	-	1.58 (0.06, 41.34)
Malaise	2 (6.89)	1 (3.57)	0	2.64 (0.12, 58.66)	1.58 (0.06, 41.34)
Arthralgia	0	1 (3.57)	0	-	1.58 (0.06, 41.34)
Myalgia	1 (3.44)	0	0	1.53 (0.06, 39.86)	-
Maculopapular rash	1 (3.44)	1 (3.57)	0	1.53 (0.06, 39.86)	1.58 (0.06, 41.34)
Eschar	0	1 (3.57)	0	-	1.58 (0.06, 41.34)
Jaundice	1 (3.44)	1 (3.57)	0	1.53 (0.06, 39.86)	1.58 (0.06, 41.34)

Comparative analysis of clinical features of SARI cases detected with viral pathogens, bacterial pathogens, and co-infection with viral and bacterial pathogens

The comparative analysis of clinical features of SARI cases detected with viral pathogen and bacterial pathogen alone versus cases involving viral-bacterial co-infection is presented in Table 5. The OR on comparing clinical features of SARI cases detected positive for viral pathogen with viral-bacterial co-infection was 0.65 (95% CI 0.44-0.97, I^2^ = 0%, p = 0.04), while the OR on comparing clinical features of SARI cases detected positive for bacterial pathogen with viral-bacterial co-infection was 0.54 (95% CI 0.38-0.76, I^2^ = 0%, p = 0.05).

**Table 5:** Comparative analysis of clinical features of SARI cases detected with viral pathogens, bacterial pathogens, and viral-bacterial co-infection.

Clinical features	Viral (n = 40) (%)	Bacterial (n = 51) (%)	Viral-bacterial Co-infection (n = 26) (%)	OR (95% CI), viral vs. co-infection	OR (95% CI), bacterial vs. co-infection
Fever	27 (67.5)	34 (66.66)	19 (73.07)	0.77 (0.26, 2.28)	0.74 (0.26, 2.09)
Chills	0	1 (1.96)	0	-	1.57 (0.06, 39.99)
Rigor	0	0	0	-	-
Rhinorrhea	10 (25)	12 (23.52)	7 (26.92)	0.90 (0.29, 2.78)	0.84 (0.28, 2.46)
Sore throat	8 (20)	13 (25.49)	6 (23.07)	0.83 (0.25, 2.76)	1.14 (0.38, 3.46)
Cough	28 (70)	33 (64.7)	19 (73.07)	0.86 (0.29, 2.58)	0.68 (0.24, 1.91)
Breathlessness	16 (40)	28 (54.9)	19 (73.07)	0.25 (0.08, 0.72)	0.45 (0.16, 1.25)
Wheezing	32 (80)	18 (35.29)	18 (69.23)	1.78 (0.57, 5.54)	0.24 (0.09, 0.67)
Crepitation	19 (47.5)	10 (19.6)	20 (76.92)	0.27 (0.09, 0.82)	0.07 (0.02, 0.23)
Headache	0	0	0	-	-
Irritability	1 (2.5)	4 (7.84)	1 (3.84)	0.64 (0.04, 10.72)	2.13 (0.23, 20.07)
Altered sensorium	1 (2.5)	2 (3.92)	1 (3.84)	0.64 (0.04, 10.72)	1.02 (0.09, 39.99)
Seizure	1 (2.5)	2 (3.92)	0	2.01 (0.01, 5.35)	2.68 (0.12, 57.82)
Abdominal pain	0	1 (1.96)	0	-	1.57 (0.06, 39.99)
Vomiting	0	2 (3.92)	1 (3.84)	0.21 (0.01, 5.35)	1.02 (0.09, 11.80)
Diarrhea	0	0	0	-	-
Malaise	0	1 (1.96)	0	-	1.57 (0.06, 39.99)
Arthralgia	0	0	0	-	-
Myalgia	0	0	0	-	-
Maculopapular rash	0	0	0	-	-
Eschar	0	0	0	-	-
Jaundice	1 (2.5)	2 (3.92)	1 (3.84)	0.64 (0.04, 10.72)	1.02 (0.09, 11.80)

Seasonal distribution of viral infection, bacterial infection, and co-infection involving both pathogens in ARI/SARI cases

The highest number of viral pathogens were detected in October (overall = 32 (16.74%); ARI = 22 (28.20%); SARI = 13 (9.92%)), followed by September (overall = 23 (11.00%); ARI = 11 (14.10%); SARI = 12 (9.16%)), while the lowest number of positive viral pathogens were detected in April (1, 2.8%) in ARI-diagnosed patient. On the contrary, no viral pathogen was detected in ARI/SARI cases in February, March, May, and June.

The highest number of bacterial pathogens was detected in September (overall = 21 (10.04%); ARI = 05 (6.41%); SARI = 16 (12.21%)), followed by December (overall = 15 (7.17%); ARI = 02 (2.56%); SARI = 13 (9.92%)). The lowest number of bacterial pathogens was detected in June (1, 1.28%) in an ARI-diagnosed patient. Other months reported a low positivity rate of bacterial pathogens, whereas no positive cases were detected in January and February in ARI/SARI cases.

The highest number of viral-bacterial co-infections was detected in September (overall = 12 (5.74%); ARI = 04 (5.12%); SARI = 08 (6.10%)), followed by October (overall = 10 (4.78%); ARI = 07 (8.97%); SARI = 03 (2.29%)), while no positive cases with viral-bacterial co-infection were detected from January to July in ARI/SARI cases.

**Figure 4:**
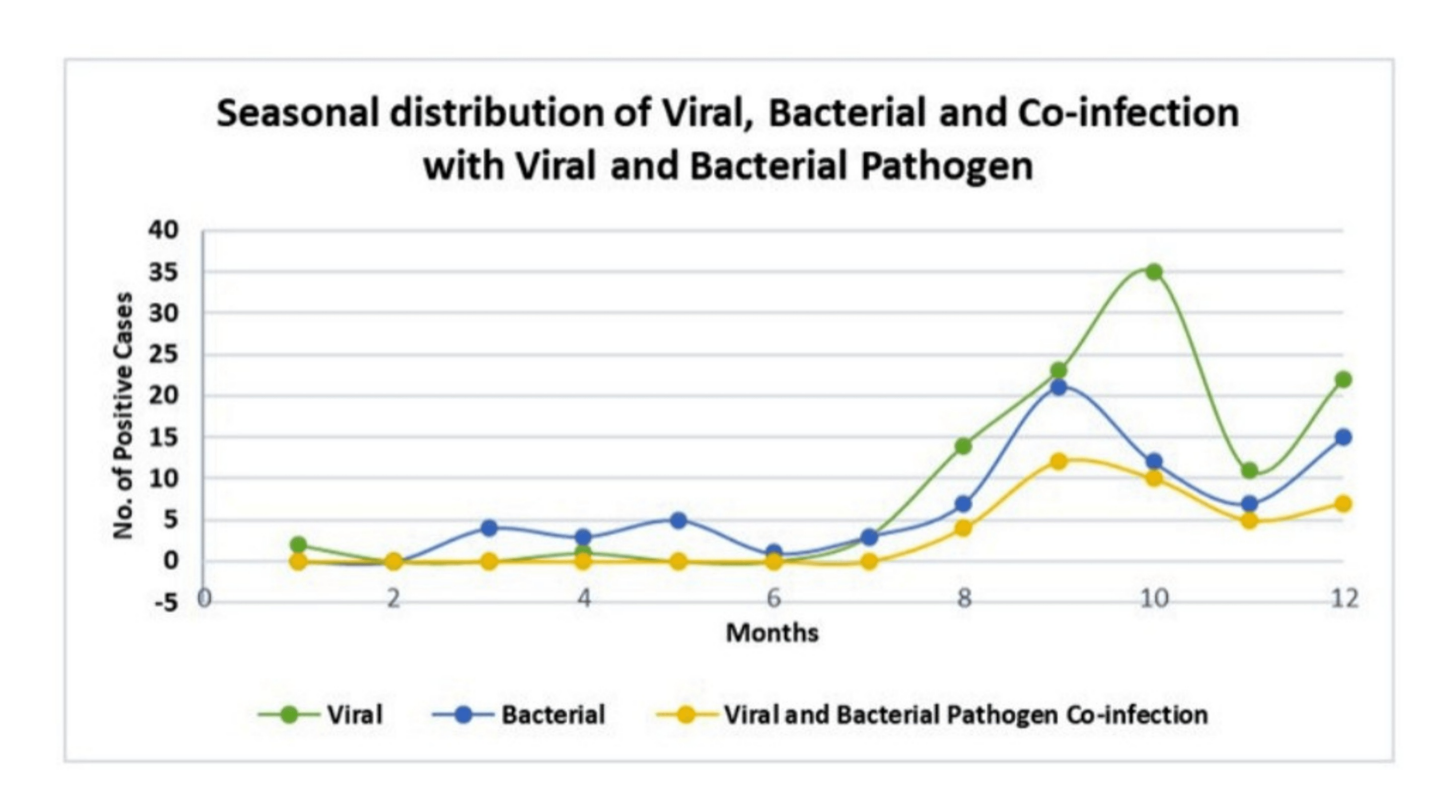
Seasonal distribution of overall positive cases detected with viral infection, bacterial infection, and co-infections involving both pathogens.

Figure 4 illustrates the seasonal distribution of overall positive cases detected with viral infection, bacterial infection, and co-infections involving both pathogens.

Comparative analysis of clinical features presented by individual viral pathogens in ARI/SARI cases

**Figure 5:**
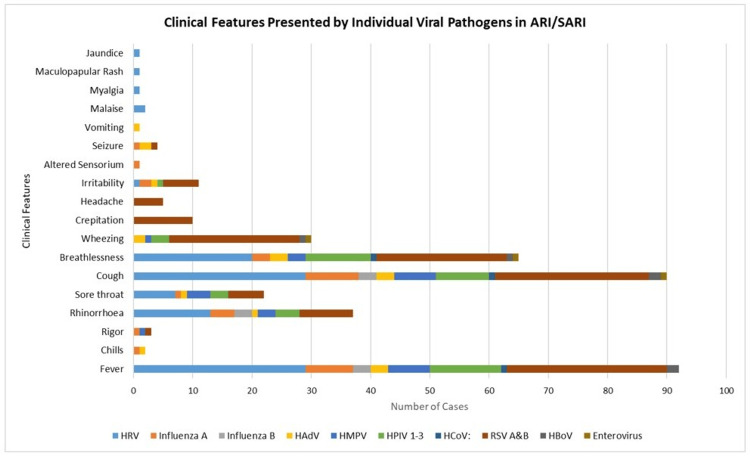
Clinical features presented by individual viral pathogens in ARI/SARI cases. ARI: acute respiratory infections, SARI: severe acute respiratory infections, HRV: human rhinovirus, RSV A and B: respiratory syncytial virus, HPIV: human parainfluenza virus, HMPV: human metapneumovirus, HAdV: human adenovirus, HBoV: human bocavirus, HCoV: human coronavirus.

In ARI/SARI, malaise (2, 4.54%), myalgia (1, 2.2%%), irritability (1, 2.2%%), and maculopapular rashes (1, 2.2%) were noticed in HRV-positive cases, while in cases positive for influenza A, altered sensorium (1, 7.69%) and seizure (1, 7.69%) was recorded in a small number of cases along with common symptoms of ARI. Among the three HAdV-positive cases, seizures were present in two (66.66%) cases, whereas irritability and vomiting were seen in one (33.33%) case. Of the two HBoV-positive cases, fever and cough were detected in both patients, whereas breathlessness and wheezing were seen in one patient. One enterovirus-positive case was detected, and the patient presented with cough, breathlessness, and wheezing. Figure 5 illustrates the clinical features presented by individual viral pathogens in ARI/SARI cases.

Comparative analysis of clinical features presented by individual bacterial pathogens in ARI/SARI cases

**Figure 6:**
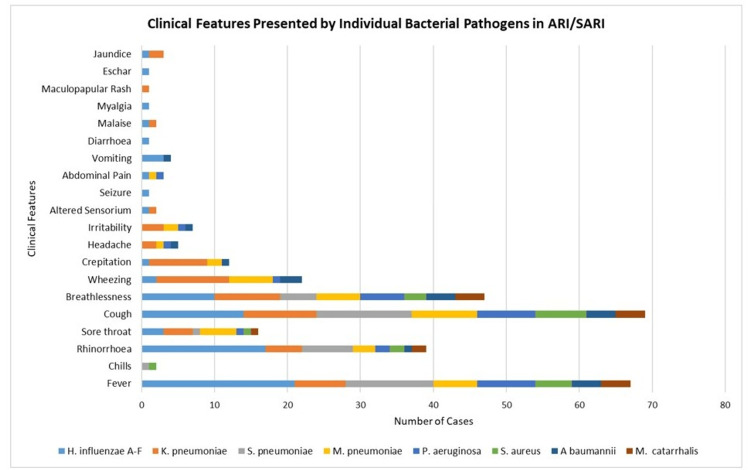
Clinical features presented by individual bacterial pathogens in ARI/SARI cases. ARI: acute respiratory infections, SARI: severe acute respiratory infections.

In ARI/SARI, out of all *H. influenzae-*positive cases, a small number of cases presented with crepitation (1, 4.37), altered sensorium (1, 4.37), seizure (1, 4.37), abdominal pain (1, 4.37), diarrhea (1, 4.37), malaise (1, 4.37), myalgia (1, 4.37), eschar (1, 4.37), and jaundice (1, 4.37). In *A. baumanii*-positive cases, along with common respiratory symptoms, crepitation (1, 16.66), headache (1, 16.66), irritability (1, 16.66), and vomiting (1, 16.66) were also seen. Figure 6 illustrates the clinical features presented by individual bacterial pathogens in ARI/SARI cases.

Clinical outcomes in relation to etiological factors and host factors

Co-infection with both viral and bacterial pathogens was detected in 40 patients with different co-infection combinations, HRV being the common virus and *H. influenzae* the common bacteria found in the combinations. On analyzing clinical outcomes with mortality and mean duration of hospitalization, co-infection with viruses and bacteria showed the highest mortality rate (5%), whereas infection with multiple viral and bacterial infections had no recorded mortality. The longest average hospitalization was observed in patients with single bacterial infections (12.42 ± 13.59 days) and multiple viral infections (12.27 ± 10.39 days). In terms of age distribution, infants aged 0-2 months exhibited the highest mortality (8.33%) and significantly longer hospital stays (30 ± 13.88 days) compared to older age groups. Male patients had a slightly higher mortality rate (2.02%) and longer hospitalization (13.45 ± 18.21 days) compared to females, as illustrated in Table 6.

**Table 6:** Clinical outcomes in relation to etiological factors and host factors.

Features	Mortality (%)	Mean duration of hospitalization
Mono-viral infection (n = 111)	1 (0.8)	10.82 ± 17.42
Poly-viral infection (n = 14)	0	12.27 ± 10.39
Mono-bacterial infection (n = 77)	1 (1.05)	12.42 ± 13.59
Poly-bacterial infection (n = 18)	0	10.53 ± 8.34
Co-infection with virus and bacteria (n = 40)	2 (5)	10.97 ± 10.63
Age group		
0-2 months	2 (8.33)	30 ± 13.88
2 months-1 year	1 (0.89)	10.07 ± 8.62
1-3 years	0	12.52 ± 19.06
3-5 years	1 (2.85)	11.91 ± 13.94
Gender		
Male	3 (2.02)	13.45 ± 18.21
Female	1 (1.63)	12.81 ± 19.26

## Discussion

The hospital-based observational study aimed to determine the etiological agents of ARI/SARI using multiplex PCR, their seasonal distribution, and frequency of mono-infection and co-infection in children under five in Eastern India between January and December 2024. It highlighted the predominance of HRV as the primary viral agent and *H. influenzae* as the leading bacterial pathogen. The findings also revealed a notable rate of viral-bacterial co-infections (8.61%) and demonstrated distinct seasonal trends, with viral infections peaking post-monsoon and bacterial infections during the monsoon period. These results emphasize the importance of considering environmental and climatic factors in disease prevention and control strategies.

In ARI and SARI cases, various viral etiologies were noticed. In ARI, RSV (24.35%) was responsible for a higher positivity rate, followed by HRV (14.1%) and influenza A (10.25%), whereas in SARI, HRV was the major etiological agent, followed by RSV (11.45%) and HPIV-1-4 (7.63%). In a study conducted in Uttar Pradesh in children under five, HPIV (11.13%) and HAdV (8.7%) were the predominant causative agents of respiratory illness, while in the present study, HPIV-1-4 and HAdV were responsible for only 7.17% and 1.43% of the infection, respectively [20]. Thirteen (9.5%) cases (median age: two years) tested positive for HCoV in a study conducted by Das et al. [21] in a tertiary care teaching hospital in Eastern India, while in our study, only one (0.47%) patient tested positive for HCoV. Another study from Rajasthan reported HMPV (25.7%) and influenza A (19.9%) as the predominant viral pathogens in SARI cases, while in the present study, HMPV and influenza A positivity rates were 6.10% and 3.81%, respectively [2]. On the contrary, in a study conducted in South Indian children under five, RSV (45.69%) was the most common viral etiological agent, followed by HRV (17.88%), whereas in our study, HRV (21.05%) contributed maximally to the overall positivity rate, followed by RSV A and B (16.26%) [22]. In a previous study conducted by our department between 2019 and 2022 in children under three, comorbid conditions, such as low birth weight, cholestatic jaundice, and pancytopenia, were found to be associated with HBoV infections, whereas in the present study, no such comorbidities were diagnosed in HBoV-positive cases [23]. Similarly, in another study carried out by our department between 2019 and 2023, HRV was the most common viral pathogen in pediatric acute LRTI cases, with a prevalence of 24.1%, whose findings are similar to the findings of the present study, where HRV is the most common viral pathogen detected [24]. The highest viral co-infection in the study was reported between HRV and RSV A and B in four (1.91%) cases, HRV and HMPV in two (0.95%) cases, and HRV and H1N1 variant of influenza A in two (0.95%) cases, which is lower than the co-infection rate of HRV and H1N1 variant of influenza A in a study conducted by Esper et al. [25]. A recently published literature by Grizer et al. [26] suggests that enterovirus can cause severe respiratory illness and paralysis in children under 16, with the greatest proportion of cases in children under five. In comparison, in our study, one (0.76%) enterovirus-positive case was detected in a SARI-diagnosed patient presenting with cough, breathlessness, and wheezing [26]. The variation in positivity rates and etiological distribution of respiratory pathogens across different studies may be due to differences in study design, sample size, and demographic characteristics. Climatic and geographical factors, such as temperature, humidity, and rainfall, also influence viral circulation and bacterial persistence. Additionally, differences in the study period, population density, and healthcare-seeking behavior can contribute to such variability.

*H. influenzae* was the chief bacterial agent, accountable for 11.53% and 10.68% positivity rates in both ARI and SARI cases, respectively. *P. aeruginosa* (31.2%) and *K. pneumoniae* (21.3%) were the predominant bacterial pathogens in respiratory samples in a previously published study, while in the present study, *P. aeruginosa *and *K. pneumoniae *accounted for only 4.78% and 7.65% of cases, respectively [27]. The predominance of *P. aeruginosa* and *K. pneumoniae* in their study could be due to the inclusion of patients with either underlying chest disease or patients with ventilator-acquired pneumonia and hospital-acquired pneumonia. In our study, of the six *A. baumannii-*positive cases, the duration of hospitalization ranged from 9 to 37 days in four cases, whereas in 10 *P. aeruginosa-*positive cases, the duration of hospitalization ranged from 9 to 37 days in six cases. The case that had hospitalization for 37 days was positive for *A. baumannii* and *P. aeruginosa*, along with *K. pneumoniae*. In the present study, the odds of infection by viral pathogen were significantly higher, compared to the odds of infection by bacterial pathogen in the case of both ARI- and SARI-diagnosed patients, with an adjusted OR of 0.91 (95% CI 0.55-1.48, I^2^ = 0%, p = 0.69) and 0.65 (95% CI 0.44-0.97, I^2^ = 0%, p = 0.04), respectively.

Knowledge related to seasonality and transmission of viral pathogens can help reduce antibiotic overdose and curb infection through timely vaccination [28]. Eastern India has summer from March to June, rainy season from July to September, and winter from October to February. The rainy season in India is common for respiratory illness [29]. From this study, it was observed that only viral mono-infections in ARI/SARI peaked in October, whereas the bacterial mono-infections and the viral-bacterial co-infections peaked in September. Additionally, the ARI cases were virus-free from January to June, with one positive case in April. Similarly, in SARI, only two positive cases were found in January. The study also reported that the viral-bacterial co-infection cases appeared around August. It was also observed that infants aged 0-2 months had the highest fatality rate (8.33%) and the longest mean hospital stay (30 ± 13.88 days), indicating a more severe disease. However, mortality dropped significantly in older age groups. In our study, males had a higher death rate than females, and their mean hospitalization length was also slightly higher. A similar trend was reported by Hasan et al., where there is higher ARI prevalence among male children across several Indian states, attributing it to both biological vulnerability and sociocultural healthcare preferences [30].

The limitation of this study is that it is a unicentric tertiary care hospital-based study conducted for only one year, with a limited sample size. Distinguishing between active infection and colonization when using syndromic multiplex PCR on samples from non-sterile body sites is not straightforward and was not conclusively addressed in this study, underscoring the importance of diagnostic stewardship [31]. Moreover, the lack of multivariable analysis precludes adjustment for potential confounders, which may have influenced the observed associations.

## Conclusions

The findings of the study indicate that HRV and *H. influenzae* were the most frequently detected pathogens among children under five with ARI/SARI in Eastern India. Climate and environment influenced both viral-bacterial co-infections and the seasonal peaks for mono-infections. The study results necessitate timely public health interventions, including increased surveillance, multiplex PCR for early detection, and investment in RSV, influenza, and *H. influenzae* vaccinations in the future. The current study lays the groundwork for community-based research to improve ARI preventive and treatment protocols and reduce ARI morbidity and death in the vulnerable pediatric population.
